# Endoscopic Ultrasound-Guided Gallbladder Drainage versus Percutaneous Gallbladder Drainage for Acute Cholecystitis: A Systematic Review and Meta-Analysis

**DOI:** 10.3390/diagnostics13040657

**Published:** 2023-02-09

**Authors:** Umesha Boregowda, Millie Chen, Shreyas Saligram

**Affiliations:** Division of Gastroenterology and Advanced Endoscopy, University of Texas Health, San Antonio, TX 78229, USA

**Keywords:** endoscopic ultrasound-guided gallbladder drainage, percutaneous gallbladder drainage, meta-analysis, acute cholecystitis, gallbladder drainage, EUS guided gallbladder drainage, lumen apposing metal stent, plastic stent

## Abstract

**Background:** Percutaneous transhepatic gallbladder drainage (PT-GBD) has been the treatment of choice for acute cholecystitis patients who are not suitable for surgery. The effectiveness of endoscopic ultrasound-guided gallbladder drainage (EUS-GBD) as an alternative to PT-GBD is not clear. In this meta-analysis, we have compared their efficacy and adverse events. **Methods:** We adhered to the PRISMA statement to conduct this meta-analysis. Online databases were searched for studies that compared EUS-GBD and PT-GBD for acute cholecystitis. The primary outcomes of interest were technical success, clinical success, and adverse events. The pooled odds ratio (OR) with a 95% confidence interval (CI) was calculated using the random-effects model. **Results:** A total of 396 articles were screened, and 11 eligible studies were identified. There were 1136 patients, of which 57.5% were male, 477 (mean age 73.33 ± 11.28 years) underwent EUS-GBD, and 698 (mean age 73.77 ± 8.7 years) underwent PT-GBD. EUS-GBD had significantly better technical success (OR 0.40; 95% CI 0.17–0.94; *p* = 0.04), fewer adverse events (OR 0.35; 95% CI 0.21–0.61; *p* = 0.00), and lower reintervention rates (OR 0.18; 95% CI 0.05–0.57; *p* = 0.00) than PT-GBD. No difference in clinical success (OR 1.34; 95% CI 0.65–2.79; *p* = 0.42), readmission rate (OR 0.34; 95% CI 0.08–1.54; *p* = 0.16), or mortality rate (OR 0.73; 95% CI 0.30–1.80; *p* = 0.50) was noted. There was low heterogeneity (I^2^ = 0) among the studies. Egger’s test showed no significant publication bias (*p* = 0.595). **Conclusion:** EUS-GBD can be a safe and effective alternative to PT-GBD for treating acute cholecystitis in non-surgical patients and has fewer adverse events and a lower reintervention rate than PT-GBD.

## 1. Introduction

Cholecystectomy is the standard of treatment for acute cholecystitis [1]. Emergent cholecystectomy is performed in up to 52.7% of the patients [2]. However, cholecystectomy is not possible in all patients with acute cholecystitis. An emergent cholecystectomy can lead to significant morbidity (up to 41%) and mortality in patients who are poor surgical candidates with severe comorbidities [3]. When cholecystectomy is not possible, percutaneous gallbladder drainage (PT-GBD) may be considered to decompress the gallbladder by placement of a cholecystostomy tube. The procedure involves placement of a catheter into the gallbladder under ultrasound or Computed tomography (CT) guidance. The technical success rate of PT-GBD ranges from 90–100%, with a clinical response rate of 56–100% [3]. However, the procedure involves a significant peri-procedure and post-procedure complication rate and morbidity. Complications (bile leak, peritonitis, bleeding, and pneumothorax) are reported in up to 10% of the patients. Patients are left with an external tube that is susceptible to dislodge, cause bleeding, pain, and increased risk of infection. Many patients would require repeated interventions and long-term management of the external drainage tube is a challenge, especially among elderly patients with extensive comorbidities and poor functional status. An alternative to PT-GBD is endoscopic ultrasound-guided gallbladder drainage (EUS-GBD). EUS-GBD is reported to have a low complication rate, length of stay, and low post-procedure pain [4]. In this technique, the gallbladder is accessed endoscopically through transmural (trans-gastric vs. trans-duodenal) stent placement. Literature suggests a technical and clinical success of EUS-GBD >90% [5,6,7].

In a meta-analysis of five studies (*n* = 495) that compared EUS-GBD and PT-GBD showed that EUS-GBD had similar technical success (OR 2.3, 95% CI 0.7–7.5, *p* = 0.18), clinical success (OR 1.1, 95% CI 0.6–2.2, *p* = 0.74) and adverse events (OR 2.9, 95% CI 0.9–9.2, *p* = 0.07. However, the reintervention rate was higher in the PT-GBD (OR 4.3, 95% CI 2.0–9.3, *p* < 0.001) [8]. One of the studies in the prior meta-analysis included patients who underwent trans-papillary gallbladder drainage rather than EUS-GBD through a transmural approach, making the results unreliable [9]. Therefore, we performed an updated meta-analysis to compare transmural EUS-GBD and PT-GBD to assess the two techniques’ efficacy and safety profile [10].

## 2. Materials and Methods

We followed the ‘Preferred reporting items for systematic review and meta-analysis’ statement (PRISMA) to conduct this meta-analysis as per the study team’s protocol [11].

### 2.1. Definitions

I. Technical success: Technical success was defined as successful placement of a drainage catheter or stent placement into the gallbladder, using PT-GBD or EUS-GBD, respectively.
II. Clinical success: Clinical success was defined as the resolution of clinical symptoms of acute cholecystitis (e.g., fever, abdominal pain, and leukocytosis) within 3 days after the procedure.
III. Re-intervention: Reintervention was defined as a repeat procedure due to stent or catheter blockage; a procedure for repositioning or replacement; or a procedure to prevent bleeding or to drain fluid.

### 2.2. Patients, Intervention, Comparison, and Outcomes (PICO)

Patients: Patients with acute cholecystitis who were not surgical candidates for cholecystectomy.
Intervention: Drainage of gallbladder through either EUS-GBD (treatment group).
Comparison: PT-GBD (control group) for the treatment of acute cholecystitis.
Outcome: Technical success, clinical success, need for reintervention, and rate of adverse events.

### 2.3. Selection Criteria

Studies that compared EUS-GBD and PT-GBD in acute cholecystitis were included. The studies published in the English language from inception to 3 December 2020, in peer-reviewed journals, were included. Studies that included patients ≥18 years of age were included, and studies of the pediatric population were excluded. We included the studies if they were prospective or retrospective cohort studies, or randomized controlled trials, that were published in a peer-reviewed journal as full-length articles or abstracts as long as they provided the data on outcome measures described below.

We excluded studies with trans-papillary gallbladder drainage, EUS-guided biliary drainage, animal studies, reviews, letters to the editor, case reports, case series with <10 patients, opinions, and studies where the data were insufficient for statistical analysis. We excluded the studies with patients age < 18 years.

### 2.4. Search Strategy and Data Extraction

A comprehensive literature search was performed on major online databases, including Embase PubMed, Cochrane Library, and Web of Science. The search period went from the study’s inception to 3 December 2020. A search for studies that compared EUS-GBD with PT-GBD for the treatment of acute cholecystitis was conducted. The following keywords and phrases were used to extract relevant studies from the electronic databases: acute cholecystitis, endoscopic ultrasound, endoscopic ultrasound-guided gallbladder drainage, EUS guided gallbladder drainage, percutaneous gallbladder drainage, trans-hepatic gallbladder drainage, cholecystostomy, and gallbladder stent, in various combinations of ‘AND’ and ‘OR.’ Further, we searched the references to identify any eligible studies based on inclusion and exclusion criteria described above.

The data collection was done by two independent authors (U.B. and M.C.) on a Microsoft Excel sheet. All the citations from the literature search were first imported to Endnote. Then, we removed the duplicate citations, followed by a screening of the articles (title and abstract) for inclusion and exclusion criteria. When there was no consensus on including a study for the pooled analysis between the two authors, a senior author (S.S.) reviewed the study personally and made the decision on whether or not the study could be included. For each study, we collected the data on the author, year of publication, country of origin, study population, gender, technical and clinical success, re-intervention rate, and adverse events for both groups.

### 2.5. Outcome Measures

The primary outcome measures of interest for the study were pooled odds ratio of technical success, clinical success, re-intervention rate, and adverse events between EUS-GBD and PT-GBD. The secondary outcomes included pooled estimates of readmission rate, procedure-related mortality, duration of the procedure, length of hospital stay, and successful cholecystectomy after gallbladder drainage. A leave-one-out sensitivity analysis was performed to assess the effect of each individual study on the pooled estimates of primary outcomes.

### 2.6. Quality Assessment

The quality of non-randomized studies was assessed with the Risk of Bias Assessment Tool for Non-Randomized Studies (ROBANs). The studies were assessed by scoring each study for the selection of study groups, comparability, and assessment of outcomes. The quality of randomized studies was assessed using the Cochrane risk of bias tool.

### 2.7. Statistical Analysis

We calculated the pooled odds ratio of technical success, clinical success, adverse events, discharge rate, and need for re-intervention. When the number of events in a group was ‘zero,’ a correction constant of 0.5 was added to calculate the pooled estimates. The pooled estimates with a 95% confidence interval were synthesized using the random-effects model, as suggested by ‘DerSimonian and Laird.’ Due to variations in the study population, study designs, and intervention, the expected effect measure may also vary, referred to as statistical heterogeneity. Heterogeneity among the included studies was assessed using the inconsistency index (I^2^). The heterogeneity was classified as low, moderate, substantial, or considerable when the I^2^ score was less than 25%, 25–50%, 50–75%, or more than 75%, respectively. Publication bias was assessed using the funnel plot and Egger’s test. Continuous variables were reported as proportions and percentages, and the categorical variables were reported as means and standard deviations.

Sensitivity analysis was performed based on the type of study (prospective vs. retrospective), type of stents used in EUS-GBD, and approach to gallbladder drainage in EUS-GBD (trans-gastric vs. trans-duodenal). Statistical analysis was performed using STATA 14.2 (Stata Corp. 4905 Lakeway Drive, College Station, TX 77845, USA).

## 3. Results

### 3.1. Search Results and Study Characteristics

**Figure 1:**
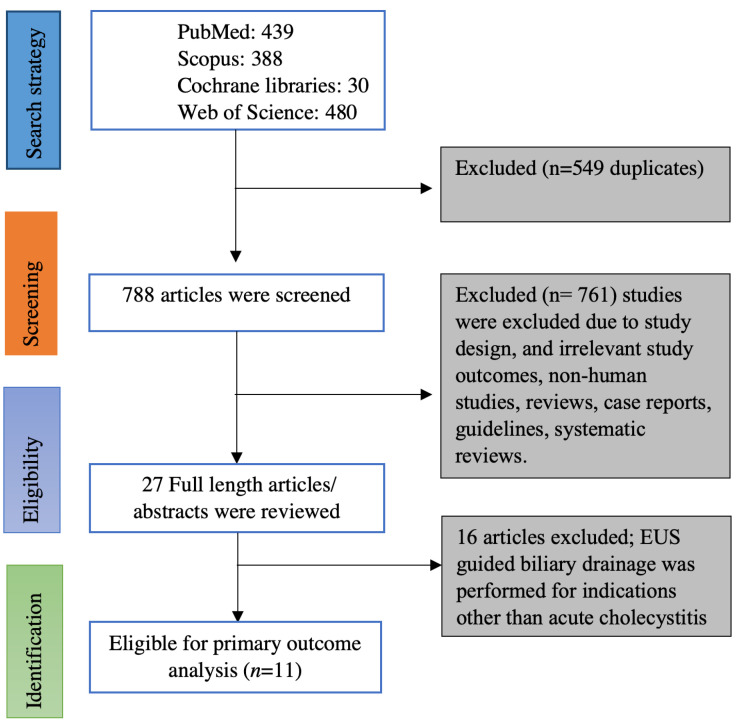
PRISMA flow chart of selecting eligible studies for the meta-analysis.

Online data search from Embase, PubMed, Web of sciences, and Cochrane database yielded 1337 studies. Duplicates were removed from the initial screening, and there was a total of 788 studies remaining for further screening. Screening the titles and abstracts yielded 27 studies for detailed review. Ultimately, we found 11 eligible studies based on inclusion and exclusion criteria [5,6,7,12,13,14,15,16,17,18,19]. Figure 1 illustrates the flow chart for the literature search and study selection. Among the 11 studies included in the meta-analysis, there were 8 retrospective studies [5,7,12,13,14,16,17,18], one of them being a propensity-matched cohort study [19] and 3 prospective studies (One prospective cohort study and two randomized controlled trials) [6,15,19]. Seven of the 11 studies were full-length articles [5,6,7,15,16,18,19] and the remaining 4 studies were published as abstracts; however, the data on outcomes were reported [12,13,14,17]. Four studies originated from the USA [13,16,17,20], two studies originated from Korea [14,15], one study from Italy [12] and Japan [19] each, and the remaining three studies were multi-center studies from more than one country [5,6,19]. Table 1 contains the characteristics of the studies included in this meta-analysis. Online literature search strategy on databases is included as a supplementary document.

### 3.2. Population and Procedure Characteristics

There were 1136 patients from 11 studies with 57.5% males. EUS-GBD was performed in 477 patients (Mean age 73.33 ± 11.28 years) and 698 (Mean age 73.77 ± 8.7 years) patients underwent PT-GBD. Although we could not calculate the exact proportions for the type of anesthesia, both IV sedation and general anesthesia were used to perform EUS = GBD; however, the Majority of the patients underwent PT-GBD under local anesthesia, need for general anesthesia was infrequent. The cause of acute cholecystitis was available from 7 full-length articles [5,6,7,15,16,18,19]. In the EUS-GBD group, 74% (275/372) of patients had calculous cholecystitis, 20% (76/372) of patients had acalculous cholecystitis, 5% (20/372) had cholecystitis due to malignancy, and 1% had other causes. In the PT-GBD group, 78% (338/431) had calculous cholecystitis, 18% (77/431) patients had acalculous cholecystitis, and 3% (14/431) had acute cholecystitis due to malignancy, and 1% had other causes. In the EUS-GBD group, a transmural gall bladder drainage through duodenum or gastric wall using various stents and drainage tubes. Lumen apposing metal stents (LAMS) was used in 287 patients, covered self-expanding metal stents (SEMS) in 92 patients, naso-biliary drainage tube in 30 patients, and plastic stents in 9 patients. One study did not report the type of stent used [19]. The choice of transmural approach for the EUS-GBD was reported by five studies (*n* = 252); 144 patients underwent trans-duodenal puncture, 106 patients had a trans-gastric puncture, and two patients had stent placement through the trans-jejunal puncture. LAMS with a diameter of 10 mm was used in 131 patients, and 15 mm LAMS was used in 62 patients (*n* = 193 from 4 studies). Data on remaining patients was not reported. In one study, patients with contraindication for PT-GBD (ascites in 5 patients and 4 patients coagulopathy/thrombocytopenia) underwent EUS-GBD [5]. There were 48 patients in the EUS-GBD group and in 32 patients in the PT-GBD group who had malignancy as an underlying comorbidity. However, cholecystitis was caused by malignancy in 20 patients among EUS-GBD group and in 14 patients among PT-GBD group [5,6,16].

### 3.3. Pooled Estimates of Primary Outcomes

**Figure 2:**
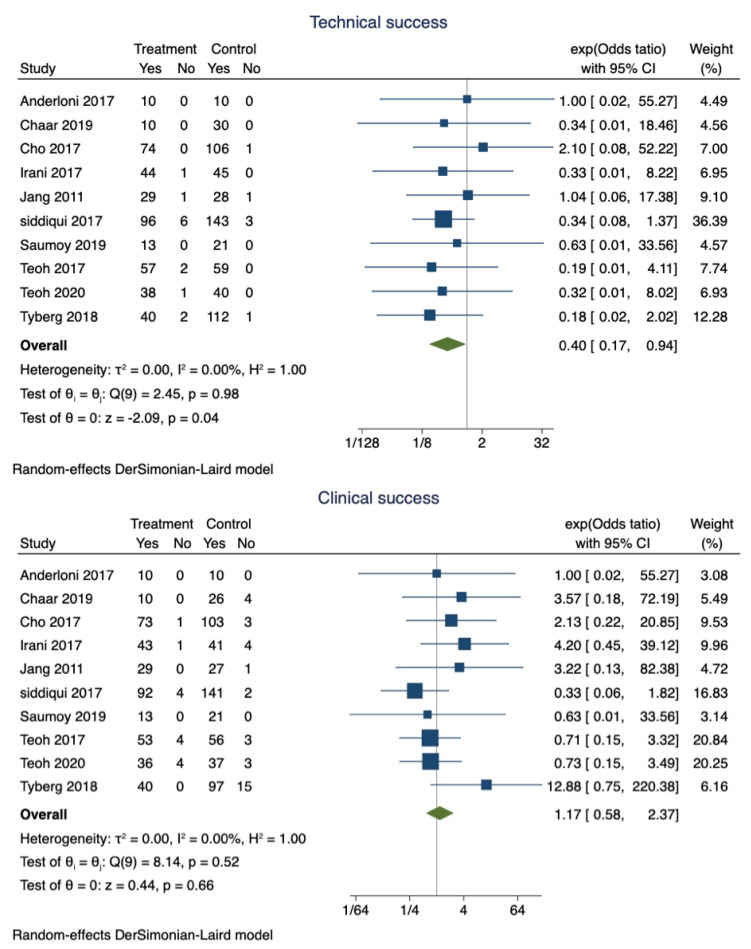
Forest plot for technical and clinical success.

**Figure 3:**
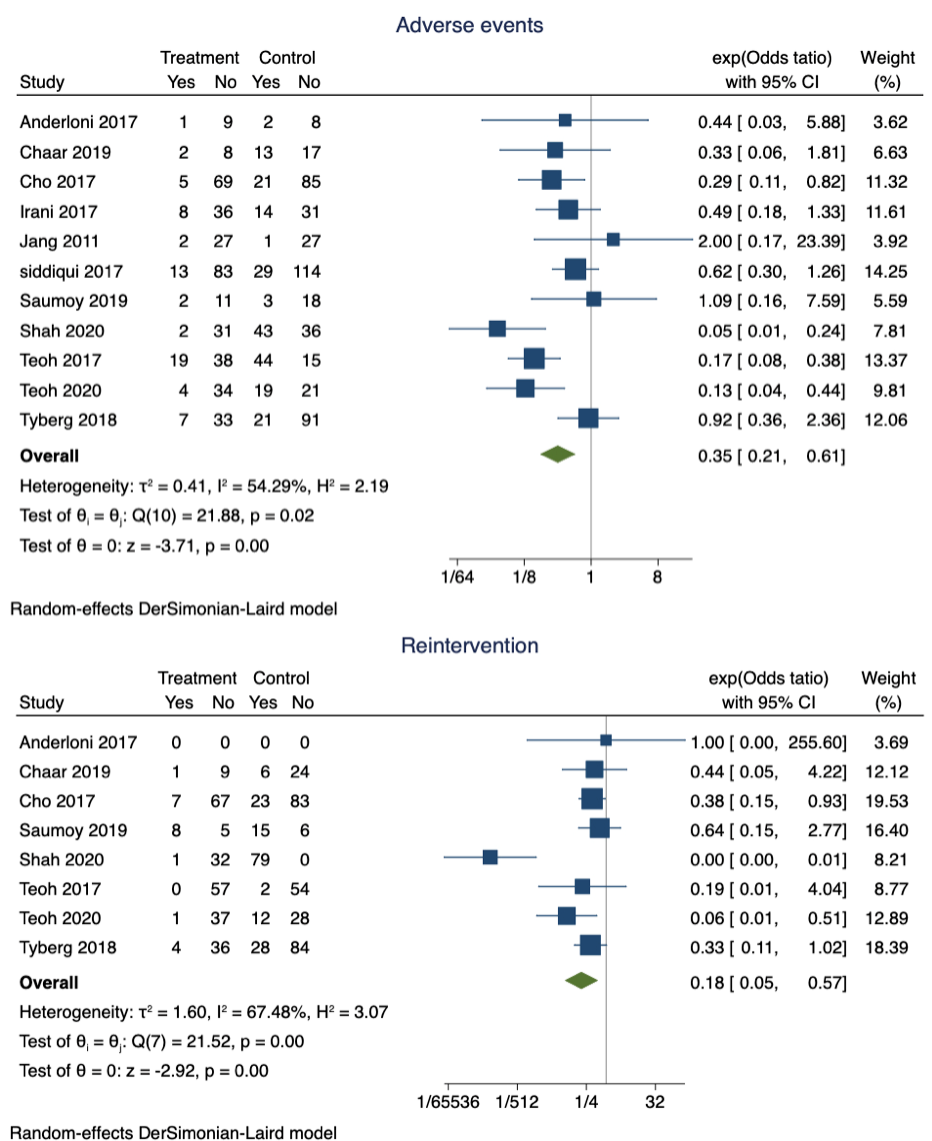
Forest plot for adverse events and reintervention rates.

- Pooled proportions and odds ratio of technical success:

EUS-GBD was successful in 89.9% of patients (95% CI 0.87–0.92), whereas PT-GBD was successful in 87.5% (95% CI 0.85–0.90). The overall pooled odds ratio of technical success was 0.40 (95% CI 0.17–0.94; I^2^ = 0; *p* = 0.04) (Figure 2). However, a sensitivity analysis excluding the studies that did not use LAMS only showed that the technical success was comparable between the two techniques (OR 0.42; 95% CI 0.15–1.15; I^2^ = 0; *p* = 0.96).

b. Pooled proportions and odds ratio of clinical success:

Clinical success was observed in 97% (95% CI 0.95–0.98) of patients who underwent EUS-GBD, and 94.1% (95% CI 0.92–0.96) of the patients who underwent PT-GBD had clinical success. The pooled odds ratio of clinical success was 1.34 (95% CI 0.65–2.79; I^2^ = 0; *p* = 0.42) (Figure 2).

c. Pooled proportions and odds ratio of adverse events:

Adverse events were noted in 14.6% (95% CI 0.11–0.18) of the patients who had successful EUS-GBD. However, nearly 30% (95% CI 0.85–0.90) of the patients who underwent PT-GBD developed adverse events. The pooled odds ratio of adverse events was 0.35 (95% CI 0.21–0.61; I^2^ = 54.29%; *p* = 0.00) (Figure 3). The data on the specific type of adverse events were not uniformly reported. Recurrent cholecystitis was noted in 7 patients who underwent EUS-GBD (7/162) and 16 in the PT-GBD group (16/195) (OR 0.75; 95% CI 0.15–3.79; I^2^ = 55.55%; *p* = 0.73) (Supplementary Figure S1). Bleeding (10 vs. 21), drain dislodgement (3 vs. 9), bile leak (4 vs. 11), and infection (0 vs. 9) were less commonly noted in EUS-GBD patients compared to PT-GBD patients. Table 2 lists some of the common complications reported by the studies.

d. Pooled proportions and odds ratio for reintervention:

The pooled proportion of reintervention in patients who underwent EUS-GBD was 15% (95% CI 0.1–0.2), and PT-GBD, it was 67% (95% CI 0.62–0.71). The pooled odds ratio of reintervention rate was 0.18 (95% CI 0.05–0.57; I^2^ = 67.48%; *p* = 0.00) (Figure 3).

#### 3.3.1. Secondary Outcomes

- Pooled proportions and odds ratio for readmission:

The pooled proportion of readmission for EUS-GBD was 16.4% (95% CI 0.11–0.23), and that of PT-GBD was 35.5% (95% CI 0.29–0.41). The pooled odds ratio of the readmission rate was 0.34 (95% CI 0.08–1.54; I^2^ = 85.39%; *p* = 0.16) (Supplementary Figure S2).

b. Pooled proportions and odds ratio of mortality:

The pooled proportions of mortality in the EUS-GBD (patients) group were 4% (95% CI 0.02–0.07) and 5.5% (95% CI 0.03–0.08). The pooled odds ratio of mortality was 0.73 (95% CI 0.30–1.80; I^2^ = 0; *p* = 0.50) (Supplementary Figure S3).

c. The pooled mean difference in procedure time:

The pooled mean difference in procedure time for EUS-GBD versus PT-GBD was 0.42 (95% CI −6.14, 6.98; I^2^ = 84.43%; *p* = 0.90). The two techniques were comparable (Supplementary Figure S4).

d. The pooled mean difference in length of hospital stays:

The pooled mean difference in length of hospital stay was −3.53 (95% CI −5.91, −1.15; I^2^ = 99.05%; *p* = 0.00) (Supplementary Figure S5). Two studies did not report the standard deviation for the outcome. Hence, a correction of 0.5 was used to calculate the pooled length of stay. A sensitivity analysis excluding those two studies also showed a significantly shorter length of hospital stay after EUS-GBD, −1.61 (95% CI −2.56, −0.67; I^2^ = 54.08%; *p* = 0.86) (Supplementary Figure S6).

e. Pooled proportion of cholecystectomy after successful drainage

The pooled proportion of patients who underwent cholecystectomy after successful drainage with EUS-GBD was 22.6% (95% CI 0.16–0.29), and for PT-GBD it was 27.3% (95% CI 0.22–0.33). The pooled odds ratio of cholecystectomy after successful gallbladder drainage was 0.32 (95% CI 0.08–1.32; I^2^ = 66.93%; *p* = 0.12) (Supplementary Figure S7).

#### 3.3.2. Validation of Meta-Analysis Results

- Sensitivity analysis:

The pooled effect size can be influenced by a single dominant study. Therefore, to assess the dominant effect of individual studies, we performed a leave one out sensitivity analysis of pooled estimates of technical success. There was no significant difference in the pooled estimates on the exclusion of any of the individual studies.

b. Heterogeneity:

The dispersion of pooled estimates was assessed using the confidence intervals (CI) and the inconsistency index (I^2^). The range of the dispersion of the pooled estimates can be assessed with CI, and the I^2^ estimates what proportion of the dispersion is true versus due to chance [21].

The CIs are reported in the summary Table 3 for pooled estimates. There was low heterogeneity (I^2^ = 0) among the studies included in the meta-analysis. Egger’s test showed no significant publication bias (*p* = 0.595).

Heterogeneity among the studies included for the pooled odds ratio of technical success, clinical success, adverse events, and mortality was low. However, there was considerable heterogeneity noted among the studies included in the pooled odds ratio of reintervention and readmission rates.

c. Publication bias:

The asymmetric distribution of the studies included in the meta-analysis on the funnel plot can provide an indication of whether or not there is a concern for publication bias. Egger’s regression test can tell us whether there is a significant concern for publication bias. A *p*-value of <0.05 was used to define the significance among the groups that were being compared. Visual inspection of the funnel plot (Supplementary Figure S8) and Egger’s test (*p* = 0.595) did not show concern for publication bias. Further nonparametric trim and fill analysis showed that observed pooled estimates would not be affected by the unpublished studies.

d. Quality assessment:

The Risk of Bias Assessment tool for Non-Randomized Studies (ROBANs) table for non-randomized studies assessing each study is shown in Supplementary Table S1. Cochrane risk of bias tool assessment of randomized studies is shown in Supplementary Table S2.

## 4. Discussion

In this meta-analysis we compared EUS-GBD and PT-GBD technique for decompression of gall bladder in acute cholecystitis who were not suitable for surgery. Our analysis showed that EUS-GBD has a significantly higher odds of technical success compared to PT-GBD (OR 0.40 (95% CI 0.17–0.94; I^2^ = 0; *p* = 0.04). However, further sensitivity analysis suggested the technical success was comparable between the group and the difference was statistically insignificant (OR 0.42; 95% CI 0.15–1.15; I^2^ = 0; *p* = 0.96). Clinical success was also comparable between the two techniques (OR 1.34; 95% CI 0.65–2.79; I^2^ = 0; *p* = 0.42). However, EUS-GBD had significantly lower rate of adverse events (OR 0.35; 95%CI 0.21–0.61; I^2^ = 54.29%; *p* = 0.00), reintervention rates (OR 0.18; 95% CI 0.05–0.57; I^2^ = 67.48%; *p* = 0.02), length of hospital stay (Mean difference −3.53 (95% CI −5.91, −1.15; I^2^ = 99.05%; *p* = 0.00). We did not find any difference in mortality (OR 0.73 (95%CI 0.30–1.80; I^2^ = 0; *p* = 0.50), procedure time (Mean difference 0.42 (95% CI −6.14, 6.98; I^2^ = 84.43%; *p* = 0.90), or the readmission (OR 0.34 (95%CI 0.08–1.54; I^2^ = 85.39%; *p* = 0.16)) between either of the techniques.

The practice guidelines recommend early cholecystectomy for acute cholecystitis [22]. However, advanced age, significant comorbidities, and the presence of multi-organ failure during the acute phase may increase the risk of peri-procedure complications and anesthesia-related complications and preclude patients from undergoing cholecystectomy. When the patient is not a candidate for cholecystectomy, either PT-GBD or EUS-GBD can be considered as an alternative to decompress the gallbladder.

EUS-GBD allows the creation of a fistulous tract between the gallbladder and GI tract (trans-duodenal vs. trans-gastric) using plastic or metal stents. Previously, EUS-GBD has been performed using plastic stents (technical success 100% and clinical success 100%), self-expanding metal stents (SEMS) (technical success 98.6% and clinical success 90%), and recently lumen apposing meta-stents (LAMS) have been used (technical success 91.5% and clinical success 90.1%) [23]. EUS-GBD with LAMS can be a permanent and definitive treatment for acute cholecystitis. Recent studies suggest LAMS are superior to plastic stents [5,6]. They are fully covered to reduce the bile leak, promote luminal apposition for better drainage, and have anti-migratory property [24]. The larger lumen of the LAMS allows stent intervention such as gallstone extraction [25]. The absence of an external drainage tube negates the need for drainage tube management by the patient, reduces the risk of accidental drainage dislodgement, a port of entry for infectious agents, and aesthetically more acceptable to the patients compared to PT-GBD.

The success of the procedure is operator-dependent, and EUS-GBD is a relatively newer technique; it is limited to large academic centers. EUS-GBD was commonly performed under general anesthesia; therefore, patients who are not candidates for cholecystectomy due to anesthesia-related contraindications may not be candidates for EUS-GBD either. However, EUS-GBD is now performed under conscious sedation and, therefore, may reduce the risk of anesthesia-related adverse events [5,15]. The safety of the procedure among patients with coagulopathy and ascites is not well established. Coagulopathy (due to medical comorbidities or medications), thrombocytopenia, and anti-platelet agents could be independent risk factors for bleeding after any invasive procedure. However, we could not evaluate the risk and benefit of EUS-GBD vs. PT-GBD in patients with coagulopathy and thrombocytopenia. Therefore, future clinical trials should address these factors.

After a EUS-GBD, closing the fistulous tract during laparoscopic or open cholecystectomy may be a challenge. Three of the included studies showed that 38 patients underwent successful cholecystectomy [6,15,18]. The investigators left the LAMS in place after EUS-GBD for at least 3 months to avoid bleeding and bile leak before undergoing cholecystectomy. One study reported that 79% patients in the EUS-GBD underwent laparoscopic cholecystectomy with closure of the gastric/duodenal puncture site [15]. This provides us important updates on probability of successful cholecystectomy can be performed after EUS-GBD and it is not a one way street as previously thought. However, larger studies are needed to asses safety of laparoscopic cholecystectomy after EUS-GBD.

The previous meta-analysis had shown that technical success, clinical success, and adverse events were similar in both techniques [8]. In contrast, in our study, EUS-GBD had significantly better technical success, lower adverse events, and reintervention rates. Increasing experience and improved techniques of EUS-GBD compared to older studies may be the likely reason for the improved outcomes.

The strengths of our study are a comprehensive literature search with a robust and well-defined inclusion and exclusion, the inclusion of high-quality studies and exclusion of duplicative studies, the inclusion of newer studies with a significantly higher number of patients, rigorous quality evaluation of included studies, and statistical evaluation of pooled estimates. We excluded the studies with patients who underwent EUS guided trans-papillary gallbladder drainage to minimize the heterogeneity among the studies. Heterogeneity among the studies can indicate the quality of included studies and the reliability of the pooled estimates. It should be noted that the heterogeneity was low (I^2^ = 0) in our meta-analysis. The studies included in this meta-analysis originated from various geographical locations (North America, Europe, Asia), making our results more generalizable.

However, there are limitations to our study that need to be mentioned. The most important limitation is that majority of the included studies were retrospective studies and included only one RCT. The EUS-GBD can be performed through either trans-gastric- or trans-duodenal approach. It should be noted that the experience of the endoscopist can influence the outcomes. However, we could not assess the effect of these variabilities on the pooled estimates. Although we found significantly lower adverse events in the EUS-GBD, the studies included in the meta-analysis did not clearly define the adverse events such as definition of bile leak, wound infection, the mode of diagnosis and their management. Therefore, this can affect the generalizability of our results.

## 5. Conclusions

In conclusion, our study is the most comprehensive review comparing the transmural EUS-GBD and PT-GBD for acute cholecystitis. We infer that EUS-GBD is a good alternative to PT-GBD in the treatment of acute cholecystitis when surgery is contraindicated. EUS-GBD improves technical success, reduces the need for reintervention. EUS -GBD has a better safety profile with lower adverse events. Both techniques have comparable clinical success, readmission rate, and mortality. Our results support that EUS-GBD is a safe and effective alternative to PT-GBD for acute cholecystitis in non-surgical patients. However, large randomized controlled trials with sufficient power and follow-up are required to validate our results.

## Figures and Tables

**Table 1:** Characteristics of studies and events in the treatment and control groups.

Author	Country	Type of Study	Stent Type		Follow Up Duration in Days	Patients (*n*)	Technical Success (*n*)	Clinical Success (*n*)	Adverse Events (*n*)	Reintervention (*n*)	Readmission (*n*)	Mortality (*n*)	Procedure Time (min)
Anderloni 2017 [12] *	Italy	Retrospective	LAMS	EUS-GBD	NA	10	10	10	1	NA	NA	NA	NA
				PT-GBD	NA	10	10	10	2	NA	NA	NA	NA
Chaar 2019 [13] *	USA	Retrospective	LAMS	EUS-GBD	122.3	10	10	10	2	1	2	0	0
				PT-GBD	151.6	30	30	26	13	6	8	5	0
Cho 2017 [14] *	Korea	Retrospective	LAMS	EUS-GBD	NA	74	74	73	5	7	NA	NA	NA
				PT-GBD	NA	107	106	103	21	23	NA	NA	NA
Irani 2017 [5]	International	Retrospective	LAMS	EUS-GBD	215 (1–621)	45	44	43	8	NA	NA	1	22
				PT-GBD	265 (1–1638)	45	45	41	14	NA	NA	3	28
Jang 2017 [15]	Korea	RCT	NBD	EUS-GBD	NA	30	29	29	2	NA	NA	NA	24
				PT-GBD	NA	29	28	27	1	NA	NA	NA	23
Siddiqui 2019 [18]	USA	Retrospective	LAMS	EUS-GBD	156	102	96	92	13	NA	NA	NA	NA
				PT-GBD	156	146	143	141	29	NA	NA	NA	NA
Saumoy 2019 [16]	USA	Retrospective	LAMS	EUS-GBD	NA	13	13	13	2	8	4	NA	NA
				PT-GBD	NA	21	21	21	3	15	2	NA	NA
Shah 2020 [17] *	USA	Retrospective	LAMS	EUS-GBD	NA	33	NA	NA	2	1	NA	1	NA
				PT-GBD	NA	79	NA	NA	43	79	NA	4	NA
Teoh 2017 [7]	Japan	Retrospective	LAMS	EUS-GBD	NA	59	57	53	19	NA	4	5	NA
				PT-GBD	NA	59	59	56	44	NA	42	1	NA
Teoh 2019 [19]	International	RCT	LAMS	EUS-GBD	365	39	38	36	5	1	10	3	22.7
				PT-GBD	365	40	40	37	19	12	14	4	27.4
Tyberg 2018 [6]	International	Prospective cohort study	Multiple	EUS-GBD	NA	42	40	40	7	4	6	0	NA
				PT-GBD	NA	113	112	97	21	28	27	4	NA

USA: United States; RCT: randomized controlled trials; LAMS: lumen apposing metal stents; EUS-GBD: endoscopic ultrasound gallbladder drainage; PT-GBD: percutaneous gallbladder drainage; NA: not available; NBD; naso-biliary drainage. * Abstracts only.

**Table 2:** Common complications associated with gallbladder drainage.

Complications	EUS-GBD	PT-GBD
Abdominal pain	6	47
Drain/stent dislodgement	3	28
Bleeding	10	22
Bile leak	4	11
Infection	0	9
Stent obstruction	6	9
Recurrent cholecystitis	6	8
Pericholecystic collection	2	3
Pneumonia	4	3
Bowel perforation	3	0
Pneumoperitoneum	2	0
Drainage around the catheter	0	2
Peritonitis	1	0

EUS-GBD: Endoscopic ultrasound-guided gallbladder drainage; PT-GBD: percutaneous gallbladder drainage.

**Table 3:** Summary of findings from meta-analysis.

Analysis	Number of Studies	Pooled Proportions (EUS vs. PT)	Pooled Estimates Odds Ratio or Mean Difference (95% CI)	I^2^	*p*-Value
Primary outcomes					
Technical success	10	89.9% vs. 87.5%	0.40 (0.17–0.94)	0%	*p* = 0.04
Clinical success	10	97% vs. 94.1%	1.35 (0.65–2.79)	0%	*p* = 0.42
Adverse events	11	14.6% vs. 30%	0.35 (0.21–0.61)	54.29%	*p* = 0.00
Reinterventions	5	15% vs. 67%	0.18 (0.05–0.57)	67.48%	*p* = 0.02
Secondary outcomes					
Readmission rate	5	16.4% vs. 35.5%	0.34 (0.08–1.54)	85.39%	*p* = 0.16
Mortality	6	4% vs. 5.5%	0.73 (0.30–1.80)	0%	*p* = 0.50
Procedure time	3	NA	0.42 (−6.14, 6.98)	84.43%	*p* = 0.90
Recurrent cholecystitis	3	4.3% vs. 8.2%	0.75 (0.15–3.79)	55.55%	*p* = 0.73
Length of hospital stay	5	7.4 ± 5.12 days vs. 11.3 ± 4.7 days	−3.53 (−5.91, −1.15)	99.05%	*p* = 0.00
Cholecystectomy	3	22.6% vs. 27.3%	0.32 (0.08–1.32)	66.93%	*p* = 0.12

EUS: endoscopic ultrasound; PT: percutaneous cholecystostomy; I^2^: inconsistency index; CI: confidence interval; NA: not applicable.

## Data Availability

The data and results that are not provided in the manuscript can be obtained from the corresponding author.

## Supplementary Materials

The following supporting information can be downloaded at: https://www.mdpi.com/article/10.3390/diagnostics13040657/s1, Figure S1. Forest plot for recurrent cholecystitis; Figure S2. Forest plot for readmission; Figure S3. Forest plot for mortality; Figure S4. Forest plot for pooled mean difference of procedure time; Figure S5. Forest plot for pooled mean difference of length of hospital stay; Figure S6. Forest plot for pooled mean difference of procedure time; Figure S7. Forest plot for pooled odds ratio of successful cholecystectomy after gallbladder drainage; Figure S8. Funnel plot assessing publication bias; Table S1: Risk of Bias Assessment Tool for Non-randomized Studies (RoBANS) summary of the risk of bias assessment; Table S2: Cochrane risk of bias tool for randomized studies.

## References

[1] Hirota M., Takada T., Kawarada Y., Nimura Y., Miura F., Hirata K., Mayumi T., Yoshida M., Strasberg S., Pitt H. (2007). Diagnostic criteria and severity assessment of acute cholecystitis: Tokyo Guidelines. J. Hepato Biliary Pancreat. Surg..

[2] Murray A.C., Markar S., Mackenzie H., Baser O., Wiggins T., Askari A., Hanna G., Faiz O., Mayer E., Bicknell C. (2018). An observational study of the timing of surgery, use of laparoscopy and outcomes for acute cholecystitis in the USA and UK. Surg. Endosc..

[3] Houghton P.W., Jenkinson L.R., Donaldson L.A. (1985). Cholecystectomy in the elderly: A prospective study. Br. J. Surg..

[4] Boregowda U., Umapathy C., Nanjappa A., Wong H., Desai M., Roytman M., Theethira T., Saligram S. (2018). Endoscopic ultrasound guided gallbladder drainage-is it ready for prime time?. World J. Gastrointest. Pharmacol. Ther..

[5] Irani S., Ngamruengphong S., Teoh A., Will U., Nieto J., Abu Dayyeh B.K., Gan S.I., Larsen M., Yip H.C., Topazian M.D. (2017). Similar Efficacies of Endoscopic Ultrasound Gallbladder Drainage with a Lumen-Apposing Metal Stent versus Percutaneous Transhepatic Gallbladder Drainage for Acute Cholecystitis. Clin. Gastroenterol. Hepatol..

[6] Tyberg A., Saumoy M., Sequeiros E.V., Giovannini M., Artifon E., Teoh A., Nieto J., Desai A.P., Kumta N.A., Gaidhane M. (2018). EUS-guided versus Percutaneous Gallbladder Drainage: Isn’t It Time to Convert?. J. Clin. Gastroenterol..

[7] Teoh A.Y.B., Serna C., Penas I., Chong C.C.N., Perez-Miranda M., Ng E.K.W., Lau J.Y.W. (2017). Endoscopic ultrasound-guided gallbladder drainage reduces adverse events compared with percutaneous cholecystostomy in patients who are unfit for cholecystectomy. Endoscopy.

[8] Khan M.A., Atiq O., Kubiliun N., Ali B., Kamal F., Nollan R., Ismail M.K., Tombazzi C., Kahaleh M., Baron T.H. (2017). Efficacy and safety of endoscopic gallbladder drainage in acute cholecystitis: Is it better than percutaneous gallbladder drainage?. Gastrointest. Endosc..

[9] Kedia P., Sharaiha R.Z., Kumta N.A., Widmer J., Jamal-Kabani A., Weaver K., Benvenuto A., Millman J., Barve R., Gaidhane M. (2015). Endoscopic gallbladder drainage compared with percutaneous drainage. Gastrointest. Endosc..

[10] Ahmed O., Rogers A.C., Bolger J.C., Mastrosimone A., Lee M.J., Keeling A.N., Cheriyan D., Robb W.B. (2018). Meta-analysis of outcomes of endoscopic ultrasound-guided gallbladder drainage versus percutaneous cholecystostomy for the management of acute cholecystitis. Surg. Endosc..

[11] Moher D., Liberati A., Tetzlaff J., Altman D.G., Group P. (2009). Preferred reporting items for systematic reviews and meta-analyses: The PRISMA statement. PLoS Med..

[12] Anderloni A., Fugazza A., Costa G., Ardito A., Mei S., Ceolin M., Pedicini V., Kurihara H., Repici A. (2017). Eus-guided versus percutaneous gallbladder drainage in fragile patients with acute cholecystitis: A retrospective analysis. Dig. Liver Dis..

[13] Chaar A., Zamora-Sifuentes J., Nasser A., Szpunar S., Barawi M. (2019). EUS-Guided versus Percutaneous gallbladder drainage in patients who are unfit for cholecystectomy: A community hospital setting. Gastrointest. Endosc..

[14] Cho D.H., Lee S.S., Oh D., Song T.J., Park D.H., Seo D.W., Lee S.K., Kim M.-H. (2017). EUS-Guided Gallbladder Drainage Reduces Late Adverse Event and Need for Re-Intervention Compared with Percutaneous Cholecystostomy in Patents Who Are Not Eligible for Surgery. Gastrointest. Endosc..

[15] Jang J.W., Lee S.S., Song T.J., Hyun Y.S., Park D.H., Seo D.-W., Lee S.-K., Kim M.-H., Yun S.-C. (2012). Endoscopic Ultrasound-Guided Transmural and Percutaneous Transhepatic Gallbladder Drainage Are Comparable for Acute Cholecystitis. Gastroenterology.

[16] Saumoy M., Tyberg A., Brown E., Eachempati S.R., Lieberman M., Afaneh C., Kunda R., Cosgrove N., Siddiqui A., Gaidhane M. (2019). Successful Cholecystectomy after Endoscopic Ultrasound Gallbladder Drainage Compared with Percutaneous Cholecystostomy, Can it Be Done?. J. Clin. Gastroenterol..

[17] Shah R.N., Khara H.S., Iqbal U., Confer B., Khan Y.I., Diehl D.L., Berger A.L., Bhanushali A., Widom K.A., Torres D.M. (2020). 731 Endoscopic ultrasound-guided gallbladder drainage (EUS-GB) compared to percutaneous gallbladder drainage (PC-GB) for high-risk non-surgical patients with acute cholecystitis: A large academic tertiary care center experience. Gastrointest. Endosc..

[18] Siddiqui A., Kunda R., Tyberg A., Arain M.A., Noor A., Mumtaz T., Iqbal U., Loren D.E., Kowalski T.E., Adler D.G. (2019). Three-way comparative study of endoscopic ultrasound-guided transmural gallbladder drainage using lumen-apposing metal stents versus endoscopic transpapillary drainage versus percutaneous cholecystostomy for gallbladder drainage in high-risk surgical patients with acute cholecystitis: Clinical outcomes and success in an International, Multicenter Study. Surg. Endosc..

[19] Teoh A.Y., Kitano M., Itoi T., Perez-Miranda M., Ogura T., Chan S.M., De la Serna C., Omoto S., Torres-Yuste R., Tsuchiya T. (2019). 1025 EUS-guided gallbladder drainage reduced adverse events as compared to percutaneous cholecystostomy in patients suffering from acute cholecystitis that were at high risk for cholecystectomy. a randomized controlled trial. Gastrointest. Endosc..

[20] Kunda R., Sharaiha R.Z., Siddiqui A., Tyberg A., Arain M.A., Noor A., Mumtaz T., Iqbal U., Loren D.E., Kowalski T.E. (2017). 212 Endoscopic Ultrasound-Guided Transmural Gallbladder Drainage Using Lumen-Apposing Metal Stents versus Endoscopic Transpapillary Drainage versus Percutaneous Cholecystostomy for Gallbladder Drainage in High-Risk Surgical Patients with Acute Cholecystit. Gastrointest. Endosc..

[21] Zhang L., Gerson L., Maluf-Filho F. (2018). Systematic review and meta-analysis in GI endoscopy: Why do we need them? How can we read them? Should we trust them?. Gastrointest. Endosc..

[22] Okamoto K., Suzuki K., Takada T., Strasberg S.M., Asbun H.J., Endo I., Iwashita Y., Hibi T., Pitt H.A., Umezawa A. (2018). Tokyo Guidelines 2018: Flowchart for the management of acute cholecystitis. J. Hepato Biliary Pancreat. Sci..

[23] Anderloni A., Buda A., Vieceli F., Khashab M.A., Hassan C., Repici A. (2016). Endoscopic ultrasound-guided transmural stenting for gallbladder drainage in high-risk patients with acute cholecystitis: A systematic review and pooled analysis. Surgical Endoscopy and Other Interventional Techniques.

[24] Walter D., Teoh A.Y., Itoi T., Perez-Miranda M., Larghi A., Sanchez-Yague A., Siersema P.D., Vleggaar F.P. (2016). EUS-guided gall bladder drainage with a lumen-apposing metal stent: A prospective long-term evaluation. Gut.

[25] Chan S.M., Teoh A.Y.B., Yip H.C., Wong V.W.Y., Chiu P.W.Y., Ng E.K.W. (2017). Feasibility of per-oral cholecystoscopy and advanced gallbladder interventions after EUS-guided gallbladder stenting (with video). Gastrointest. Endosc..

