# Supplementary material for: Endoscopic Ultrasound-Guided Gallbladder Drainage versus Percutaneous Gallbladder Drainage for Acute Cholecystitis: A Systematic Review and Meta-Analysis
Source: Diagnostics (Basel). 2023 Feb 9;13(4):657. doi: 10.3390/diagnostics13040657 (PMC9954901; doi:10.3390/diagnostics13040657)
Supplement: Supplementary file 1 [file diagnostics-13-00657-s001.zip › diagnostics-2063099-supplementary.pdf]

Supplementary Figure S1. Forest plot for recurrent cholecystitis.

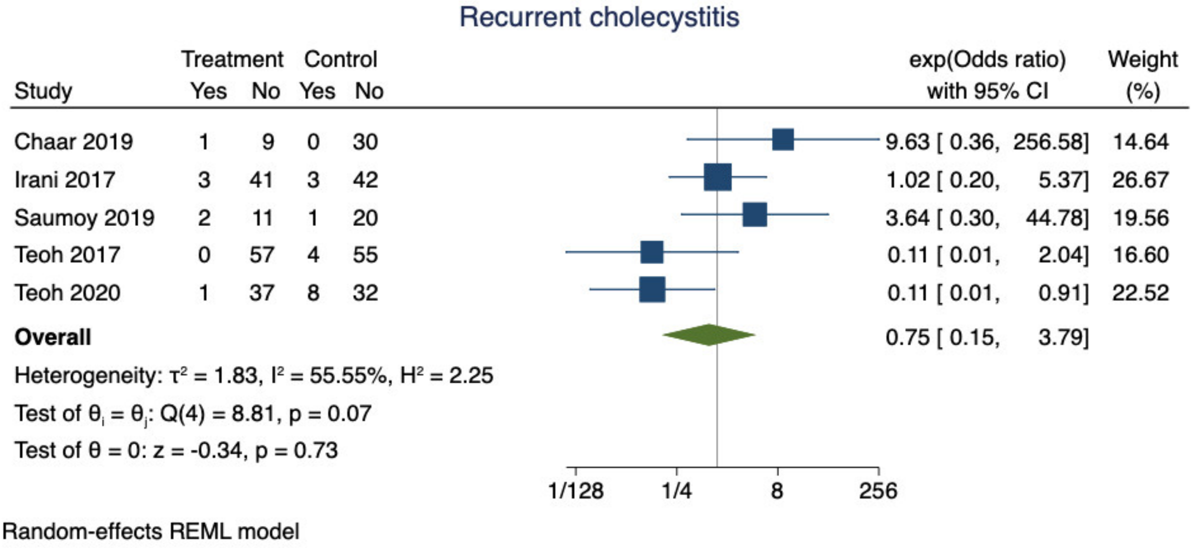

Supplementary Figure S2. Forest plot for readmission.

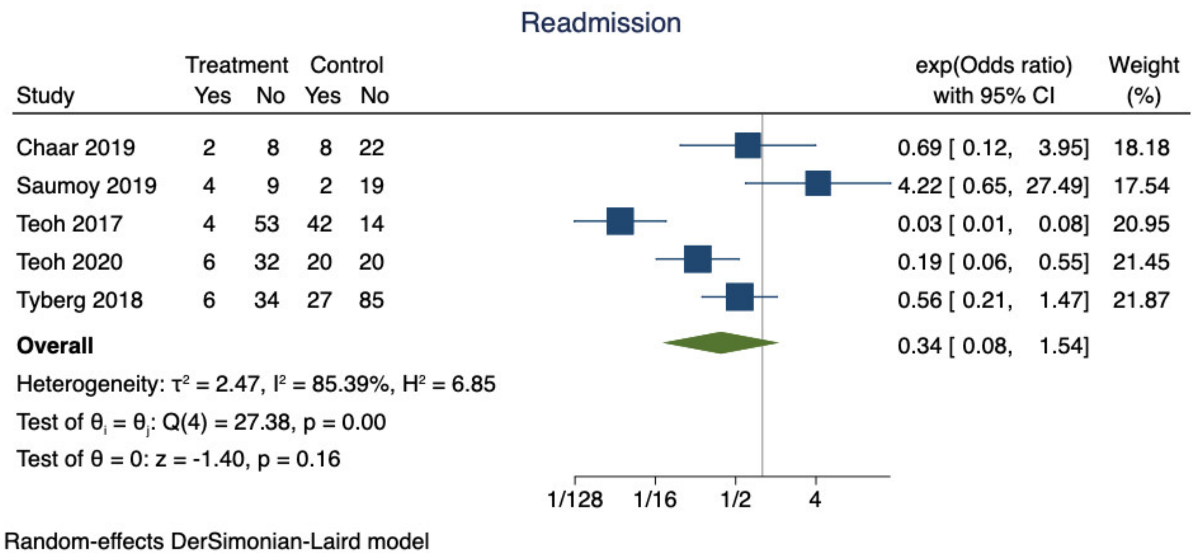

Supplementary Figure S3. Forest plot for mortality.

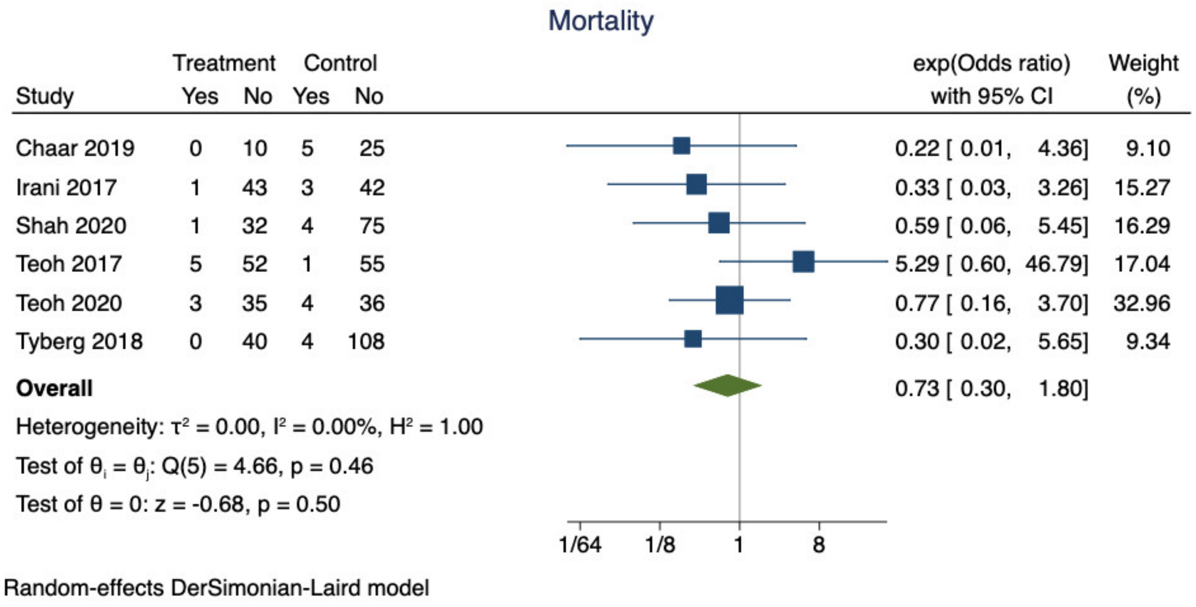

Supplementary Figure S4. Forest plot for pooled mean difference of procedure time.

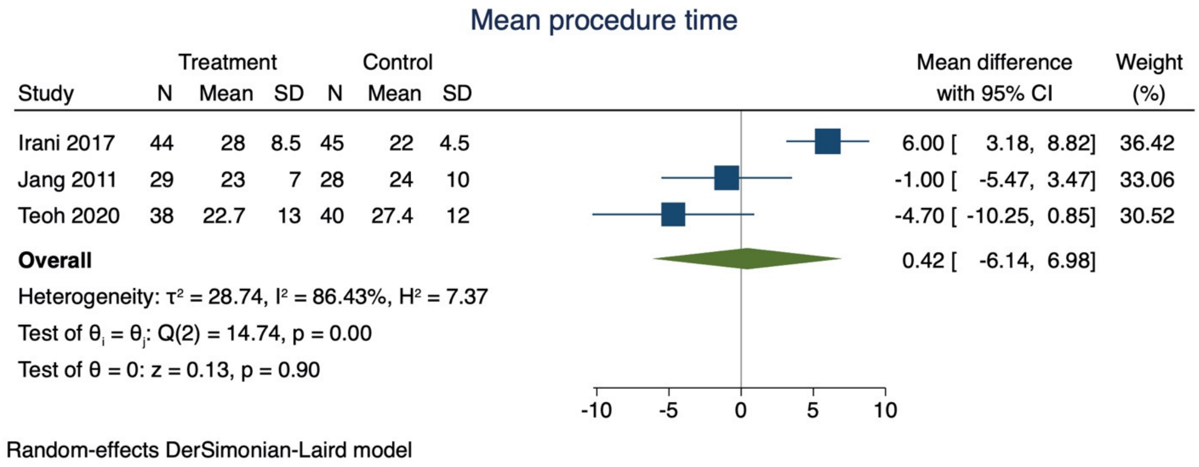

Supplementary Figure S5. Forest plot for pooled mean difference of length of hospital stay.

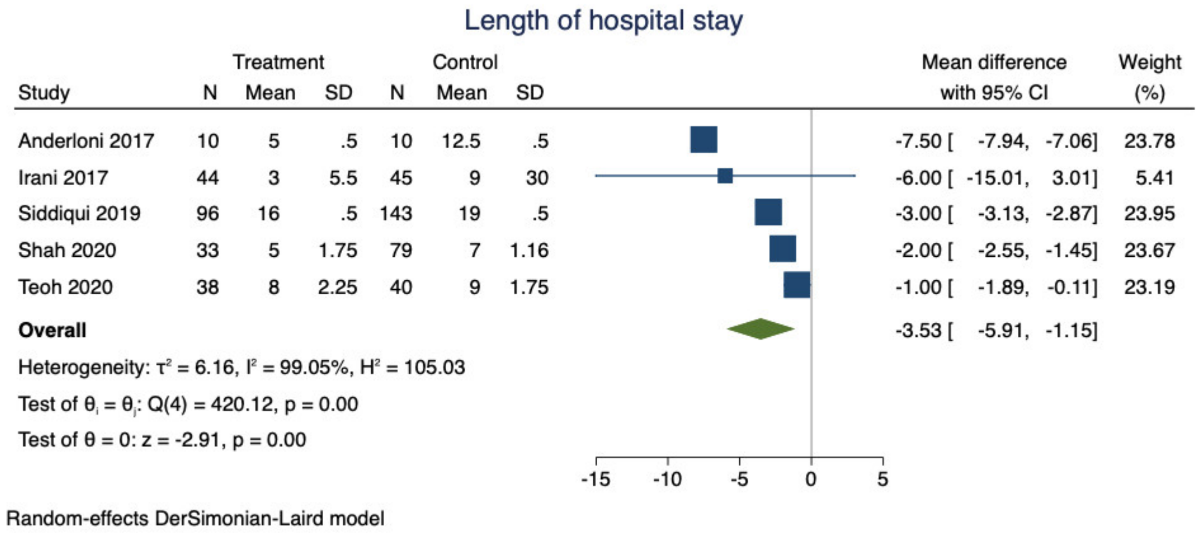

Supplementary Figure S6. Forest plot for pooled mean difference of procedure time.

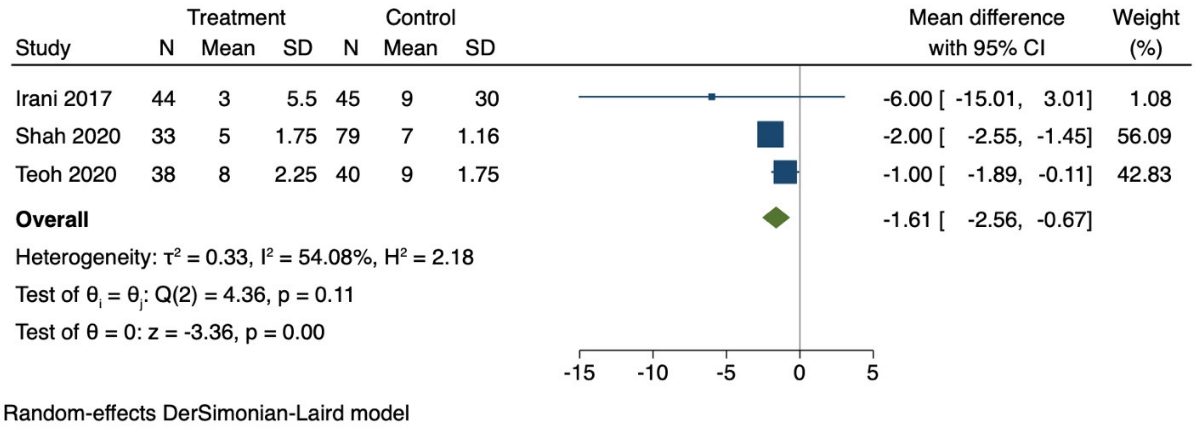

Supplementary Figure S7. Forest plot for pooled odds ratio of successful cholecystectomy after gallbladder drainage.

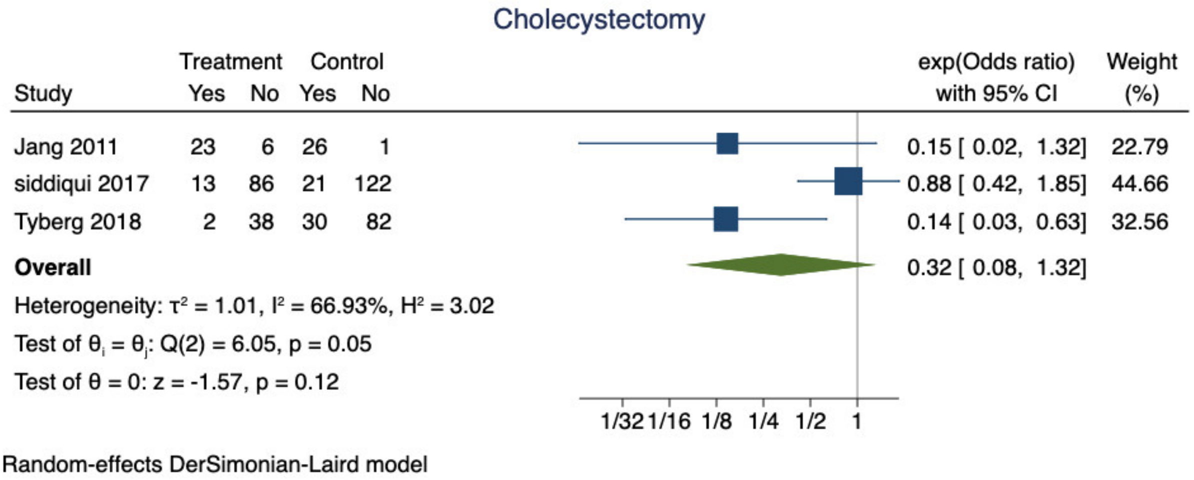

Supplementary Figure S8. Funnel plot assessing publication bias.

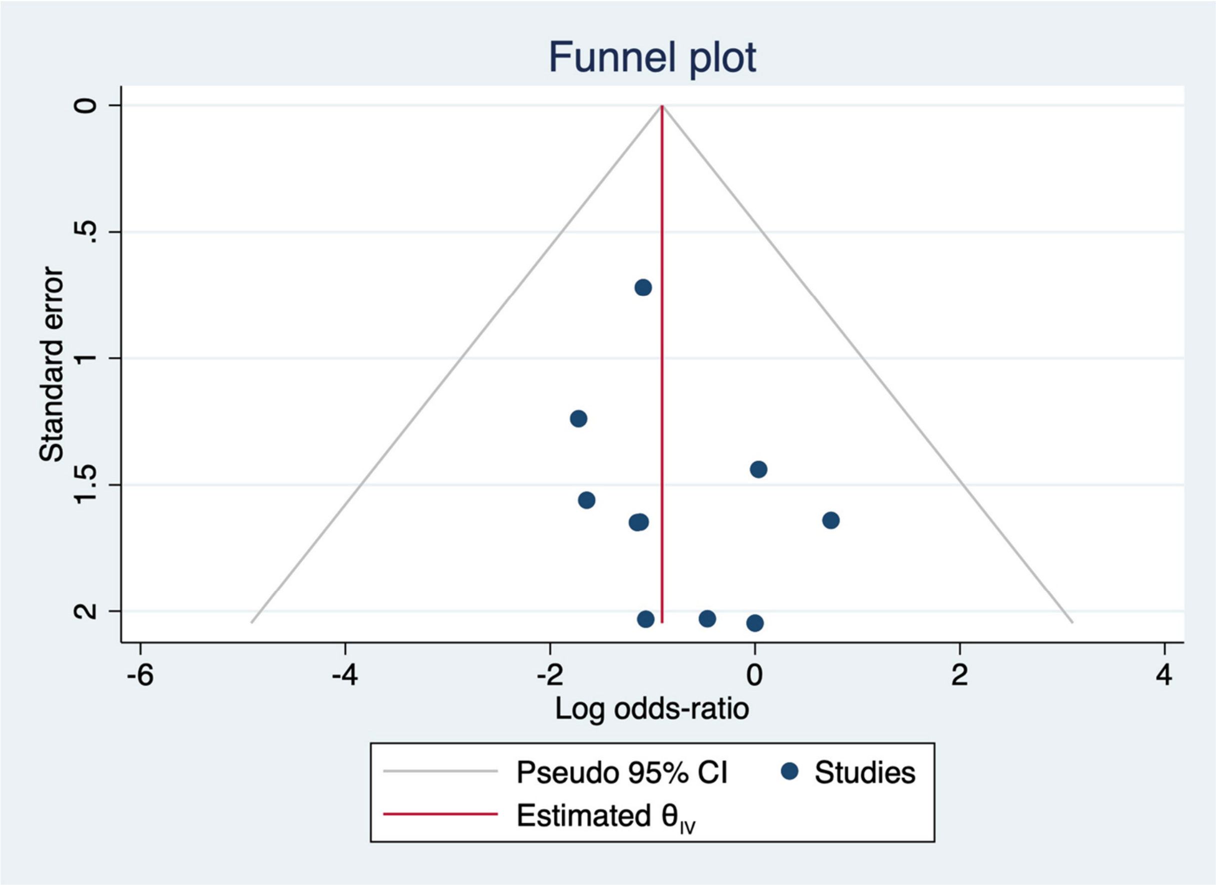

Supplementary Table S1: Risk of Bias Assessment Tool for Non-randomized Studies (RoBANS) summary of the risk of bias assessment.

	Selection		Performance	Detection	Attrition	Reporting
**Study**	**Selection of Participants**	**Confounding variables**	**Measurement of exposure**	**Blinding of outcome assessment**	**Incomplete outcome data**	**Selective outcome reporting**
Anderloni 2017 [12]	High	Low	Low	High	Low	Low
Chaar 2019 [13]	High	Low	Low	High	Low	Low
Cho 2017 [14]	High	Low	Low	High	Low	Low
Irani 2017 [5]	High	Low	Low	High	Low	Low
Siddiqui 2019 [18]	High	Low	Low	High	Low	Low
Saumoy 2019 [16]	High	Low	Low	High	Low	Low
Shah 2020 [17]	High	Low	Low	High	Low	Low
Teoh 2017 [7]	High	Low	Low	High	Low	Low

Supplementary Table S2: Cochrane risk of bias tool for randomized studies.

	Allocation concealment (selection bias)	Blinding of participants and personnel (performance bias)	Blinding of outcome bias (detection bias)	Incomplete outcome bias (attrition bias)	Selective reporting (reporting bias)	Other bias
**Jang 2011 [15]**	Low	High	High	Low	Low	Low
**Teoh 2019 [19]**	Low	High	High	Low	Low	Low
**Tyberg 2018 [6]**	High	High	High	Low	Low	Low
